# Clinical study of modular external fixator for distal humeral metaphyseal-diaphyseal junction fractures in children

**DOI:** 10.3389/fped.2026.1772321

**Published:** 2026-02-13

**Authors:** Shuzhen Deng, Zhaoqiang Chen, Zhongli Zhang, Zhe Fu, Huaguo Zhao, Jin Cao, Dan Wu, Jingyu Zhang, Yongcheng Hu

**Affiliations:** 1 Graduate School of Tianjin Medical University, Tianjin, China; 2 Department of Pediatric Orthopedics, Tianjin Hospital, Tianjin, China; 3 Department of Orthopedics, Ningbo Sixth Hospital, Ningbo, China; 4 Department of Bone and Soft Tissue Tumors, Tianjin Hospital, Tianjin, China

**Keywords:** closed reduction, distal humeral fracture, external fixation configuration, metaphyseal-diaphyseal junction, modular external fixator, pediatric fracture

## Abstract

**Background:**

This study evaluated the clinical efficacy of closed reduction with modular external fixation for pediatric distal humeral metaphyseal-diaphyseal junction (MDJ) fractures.

**Methods:**

We retrospectively analyzed 45 pediatric patients (mean age 7.87 ± 5.03 years) treated from January 2020 to December 2024. Patients were divided into “linear” (*n* = 18, two half-pins/one rod) and “triangular” (*n* = 27, four half-pins/three rods) configuration groups based on external fixator configuration. Outcomes included fracture healing time, radiographic parameters (Baumann angle, shaft-condylar angle), Flynn elbow scores, and complications. Fracture line height (The vertical distance from the highest point of the fracture line to the upper edge of the olecranon fossa) was retrospectively measured to analyze configuration selection.

**Results:**

The mean operative time was 42.8 ± 12.3 min in the “linear” group and 46.5 ± 17.9 min in the “triangular” group. All fractures healed satisfactorily. Healing time (11.0 ± 2.6 weeks vs. 10.2 ± 3.2weeks), final Baumann angles (72.80 ± 3.71 ° vs. 73.81 ± 7.40 °), and shaft-condylar angles (39.38 ± 8.32 ° vs. 35.51 ± 7.67 °) showed no significant differences between groups (*P* > 0.05). Excellent-to-good Flynn scores were achieved in 94.44% (linear) vs. 92.31% (triangular) of patients, with no statistically significant difference (*P* > 0.05). Complications included pin tract infections (7 vs. 11 cases) and fixation loosening (2 cases each). The average fracture line height was significantly lower in the “linear” configuration group (17.51 ± 3.35 mm) compared to the “triangular” group (28.02 ± 7.93 mm).

**Conclusions:**

Modular external fixation demonstrated promising outcomes for pediatric distal humeral MDJ fractures, providing a minimally invasive treatment option with favorable functional recovery, and no second surgery is required to remove the internal fixation device. Low-position MDJ fractures (<20 mm) with a linear configuration can be sufficiently stabilized with K-wires, and the “triangular” configuration is more suitable for high-level fractures (≥20 mm).

## 1 Introduction

Metaphyseal-diaphyseal junction (MDJ) fractures of the distal humerus in children, first defined by Professor Fayssoux in 2008 (1), refer to fractures occurring between the superior margin of the olecranon fossa and the proximal extent of metaphyseal widening of the distal humeral shaft (2, 3). These fractures account for approximately 3% of all supracondylar humeral fractures (1). Unlike traditional supracondylar fractures, MDJ fractures occur more proximally with reduced cross-sectional area, longer distal lever arm, and poor inherent stability, These features complicate stabilization and increase risks of treatment failure (4–6).

Currently, closed reduction and percutaneous Kirschner wire (K-wire) fixation remains the most commonly reported surgical technique for treating distal humeral MDJ fractures in children, as documented in multiple clinical series (7–9). However, K-wires often fail to provide sufficient stability for MDJ fractures due to the higher fracture line, which increases surgical time and with more complications (10). Several authors have reported the use of elastic stable intramedullary nails (ESIN) for treating pediatric distal humeral MDJ fractures (11–13), with clinical results demonstrating superior outcomes compared to traditional K-wire fixation. Nevertheless, considerable controversy remains regarding this technique. ESIN fixation in metaphyseal fractures violates the three-point fixation principle, provides only uniplanar stability, and exhibits poor resistance to rotational forces and sagittal plane stress (14, 15). Additionally, ESIN removal requires a second surgical procedure, increasing the number of operations and the risk of iatrogenic trauma.

Modular External Fixator offers unique advantages in fracture management, including the ability to insert half-pins at optimal locations intraoperatively, utilization of half-pins as joysticks to facilitate fracture reduction, flexible configuration of the external frame's spatial construct, and elimination of the need for reoperation during removal, while providing superior stability compared to K-wires. Previous literature has reported the application of external fixation for pediatric supracondylar humeral fractures (16–18), particularly for unstable, irreducible, and comminuted fractures (19). However, a comprehensive review of domestic and international literature reveals a paucity of reports on external fixation for pediatric distal humeral MDJ fractures, with existing studies predominantly focusing on biomechanical investigations (5, 6). Clinical studies remain scarce, limiting the availability of evidence-based guidance for clinical practice. Therefore, the current study retrospectively analyzed the clinical data of pediatric patients with distal humeral MDJ fractures treated with modular external fixator at the Department of Pediatric Orthopedics, Tianjin Hospital, Tianjin, China, from January 2020 to December 2024. The objectives of this study are to evaluate the clinical efficacy of this surgical approach, summarize the technical key points and explore the selection of modular external fixation configuration application.

## 2 Materials and methods

### 2.1 Study design and patient selection

This retrospective cohort study was reported in accordance with the Strengthening the Reporting of Observational Studies in Epidemiology (STROBE) guidelines.

A retrospective analysis was conducted on pediatric patients with distal humeral metaphyseal-diaphyseal junction (MDJ) fractures treated at the Department of Pediatric Orthopedics, Tianjin Hospital, Tianjin, China, from January 2020 to December 2024.

The inclusion criteria were as follows: (1) Age ≤14 years. (2) Closed distal humeral MDJ fractures without involvement of the articular surface (intercondylar region). (3) No neurovascular injury requiring open exploration. (4) Treatment with closed reduction and modular external fixation (with or without Kirschner wire supplementation). (5) Complete clinical follow-up data available.

The exclusion criteria were: (1) Age >14 years. (2) Open distal humeral MDJ fractures, fractures involving the articular surface, or concomitant fractures at other sites. (3) Neurovascular injury requiring open exploration. (4) Treatment methods other than modular external fixation. (5) Pathological fractures. (6) Incomplete clinical follow-up data.

A total of 98 patients with distal humeral fractures in the MDJ region were initially identified from our institutional database during the study period. After applying inclusion and exclusion criteria, 53 patients were excluded for the following reasons: treatment with methods other than modular external fixation (*n* = 31, including 29 treated with K-wire fixation alone and 2 treated with ESIN); age >14 years (*n* = 3); open fractures requiring surgical debridement (*n* = 4); fractures extending into the articular surface (*n* = 3); concomitant ipsilateral forearm fractures (*n* = 3); pathological fractures (*n* = 2); neurovascular injury requiring open exploration (*n* = 5); and incomplete follow-up data with loss to follow-up before fracture union confirmation (*n* = 2).

The final study cohort comprised 45 patients who met all inclusion criteria, including 28 males and 17 females. The fractures occurred on the left side in 21 patients and the right side in 24 patients. The mean age at surgery was 7.87 ± 5.03 years (range, 2.58–12.75 years). The mechanisms of injury included falls in 34 patients, falls from height in 7 patients, and traffic accidents in 4 patients. Based on the fracture classification system reported in the literature (5, 7), there were 23 transverse fractures, 12 oblique fractures, and 10 comminuted fractures.

**Figure 1:**
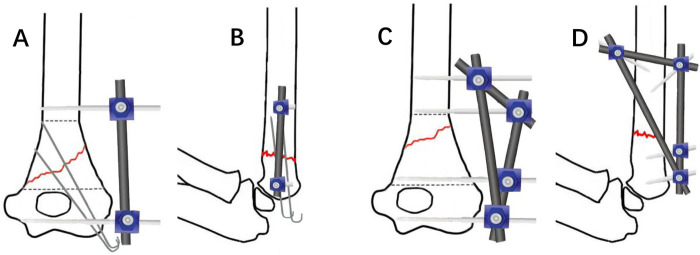
Treatment of distal humeral MDJ fractures in children, a “linear” configuration external fixator composed of two half-pins and a connecting rod, combined with K-wire fixation. **(A)** Coronal view schematic diagram, **(B)** sagittal view schematic diagram. A “triangular” configuration of an external fixator composed of four half-pins and three connecting rods, **(C)** coronal view schematic diagram, **(D)** sagittal view schematic diagram.

The treatment method involves closed reduction and fixation with a modular external fixator. According to the configuration of the external fixator, patients were divided into two groups: the “linear” configuration group (*n* = 18), consisting of two half-pins connected by one rod (Kirschner wires need to be used in combination, Figures 1A,B), and the “triangular” configuration group (*n* = 27), consisting of four half-pins connected by three rods (Figures 1C,D). Detailed information for both groups is presented in Table 1.

**Table 1:** Comparison of baseline characteristics between groups.

Group	Gender		Side		Age at surgery (years)	Type of fracture		
	Male	Female	Left	Right		Transverse	Oblique	Comminuted
Linear	11	7	8	10	7.23 ± 2.27	10	4	4
Triangular	17	10	13	14	7.27 ± 2.73	13	8	6
Statistic	*χ*^2^ = 0.16		*χ*^2^ = 0.06		*t* = −0.037			
*P* value	0.9		0.807		0.971	0.927^a^		

Data are presented as *n* or mean ± standard deviation. *χ*^2^, chi-square test; t, independent samples *t*-test.

a Fisher's exact test for categorical variables with expected cell counts <5.

### 2.2 Surgical technique

All procedures were performed by two senior pediatric orthopedic surgeons (>10 years of experience in pediatric fracture management) under general anesthesia with the patient in the supine position and the affected limb abducted. Standard sterile preparation and draping were performed. The fracture line was localized under C-arm fluoroscopy and marked on the skin surface.

In the “triangular” configuration, the two half-pins in the distal fragment are placed one below the olecranon fossa of the ulna at the metaphysis and the other approximately 1 cm above the fracture line, both inserted percutaneously from the lateral side of the elbow joint, perpendicular to the longitudinal axis of the distal humerus. The two half-pins in the proximal fragment are inserted one from the lateral side of the elbow and the other usually from the anterolateral side at approximately a 45 ° angle. To ensure that the three lateral half-pins can be fixed to a single connecting rod, the distal half-pin must be inserted with the elbow crease facing upwards, and the proximal half-pin is also inserted from the purely lateral side. All three half-pins are perpendicular to the longitudinal axis of the bone segment (Figures 1C,D). In the “linear” configuration, the distal half-pin is usually inserted into the metaphysis below the olecranon fossa of the ulna, and the proximal half-pin is inserted above the fracture line on the lateral side of the elbow. During proximal pin insertion, a drill sleeve was used throughout the procedure to protect the radial nerve. Half-pins with a diameter of 4.0 mm were used for older and heavier patients, while 3.0 mm diameter half-pins were used for younger and lighter patients.

For fracture reduction and fixation, the upper arm was placed flat on the operating table while an assistant-maintained traction. Under C-arm fluoroscopy guidance, closed reduction was achieved using the half-pins. Coronal plane angulation and displacement were corrected first. After satisfactory coronal alignment was achieved, the elbow was gradually flexed while maintaining traction, and lateral fluoroscopy was used to correct sagittal plane angulation and displacement. Once satisfactory reduction was achieved, the half-pins were secured to the connecting rods and locked. The “linear” configuration is used with two pins and one rod, K-wires must be used in combination to achieve spatial stability. The K-wires are inserted percutaneously from either the lateral or medial side of the elbow, depending on the fracture pattern. The “triangular” configuration is used with four pins and three rods, and Kirschner wires were not used if stability was adequate. The stability of the fracture site and elbow range of motion were assessed. After confirmation of satisfactory results, the wounds were irrigated. When Kirschner wires were used, they were bent and cut. The incisions were closed and dressed with sterile bandages.

### 2.3 Postoperative management and follow-up

For younger patients unable to cooperate with treatment and obese older patients, cast protection was applied for 2–3 weeks postoperatively. Other patients were encouraged to perform early active functional exercises under forearm sling protection. Regular pin site care was performed. Radiographs were obtained at 4, 8, 12, and 16 weeks postoperatively. The timing of external fixator and Kirschner wire removal was determined based on fracture healing status. During the period of external fixation, regular pin site care is necessary. If there is no infection at the pin site, the site should be disinfected and cared for regularly every three days. If a needle tract infection occurs, professional care of the needle tract is required daily, and oral antibiotics may be necessary.

All the external fixator removal was performed in the outpatient clinic without any sedation or anesthesia, using only topical anesthetic cream (EMLA: lidocaine 2.5%/prilocaine 2.5%) applied to pin sites 45 min prior to removal.

Follow-up evaluations were conducted at 6 and 12 months postoperatively to assess fracture remodeling, elbow function, and complications, with annual follow-up thereafter.

### 2.4 Data collection and outcome assessment

The following data were recorded: operative time, and postoperative follow-up duration. Fracture union was defined as blurring of the fracture line on standard anteroposterior and lateral elbow radiographs with healing of three out of four cortices, and the time to union was recorded. At the final follow-up, the Baumann angle was measured on standard anteroposterior elbow radiographs, and the shaft-condylar angle was measured on standard lateral elbow radiographs. Elbow function at the final follow-up was evaluated according to the Flynn criteria for elbow performance (20): excellent (loss of motion ≤5°), good (loss of motion 6°–10°), fair (loss of motion 11°–15°), and poor (loss of motion >15°). Complications during treatment were recorded, including cubitus varus, pin tract infection, fixation loosening, nerve injury, fracture redisplacement, and refracture.

To further facilitate clinical application, we added fracture line height as a new measurement indicator. The measurement method involves measuring the vertical distance from the highest point of the fracture line to the upper edge of the olecranon fossa of the ulna on an anteroposterior x-ray image of the elbow joint. Due to the significant fracture displacement preoperatively, which made accurate measurement difficult, we used intraoperative post-reduction x-ray images for this assessment.

All radiographic measurements were performed on a PACS workstation with our hospital by two independent senior radiologists (>10 years of experience in pediatric musculoskeletal imaging). To account for radiographic magnification, measurements were calibrated using the known diameter of the implanted half-pins (3.0 mm or 4.0 mm) as an internal reference scale. Inter-observer reliability was assessed using intraclass correlation coefficients (ICC), with ICC values >0.85 indicating excellent agreement.

### 2.5 Statistical analysis

Statistical analyses were performed using SPSS version 31.0 (SPSS Inc., Chicago, IL, USA). Continuous variables were tested for normality using the Shapiro–Wilk test. Normally distributed data (age at surgery, time to fracture union, Baumann angle, and shaft-condylar angle) were expressed as mean ± standard deviation (x¯ ± s), and comparisons between groups were performed using independent samples *t*-test. Non-normally distributed data would be analyzed using Mann–Whitney *U* test. Categorical variables (gender and side) were expressed as frequencies (*n*, %), and comparisons between groups were performed using chi-square test and Fisher's exact test. A two-tailed significance level of *α* = 0.05 was used for all statistical tests.

As this was a retrospective study, no *a priori* sample size calculation was performed. *post-hoc* power analysis using G*Power 3.1 indicated that with 45 patients (18 vs. 27), the study had 80% power to detect a medium effect size (Cohen's d = 0.5) at *α* = 0.05 for the primary outcome.

## 3 Results

### 3.1 Study selection and characteristics

There were no statistically significant differences between the “linear” configuration group and the “triangular” configuration group in terms of gender, laterality, age at surgery, or initial fracture type (*P* > 0.05, Table 1).

All patients underwent successful surgical procedures without intraoperative complications. The mean operative time was (42.8 ± 12.3) min in the “linear” configuration group and (46.5 ± 17.9) min in the “triangular” configuration group. The mean follow-up period was 29.7 months (range: 9–45 months) in the “linear” configuration group and 31.3 months (range: 7–48 months) in the “triangular” configuration group.

### 3.2 Main outcomes

#### 3.2.1 Radiographic outcomes

**Figure 2:**
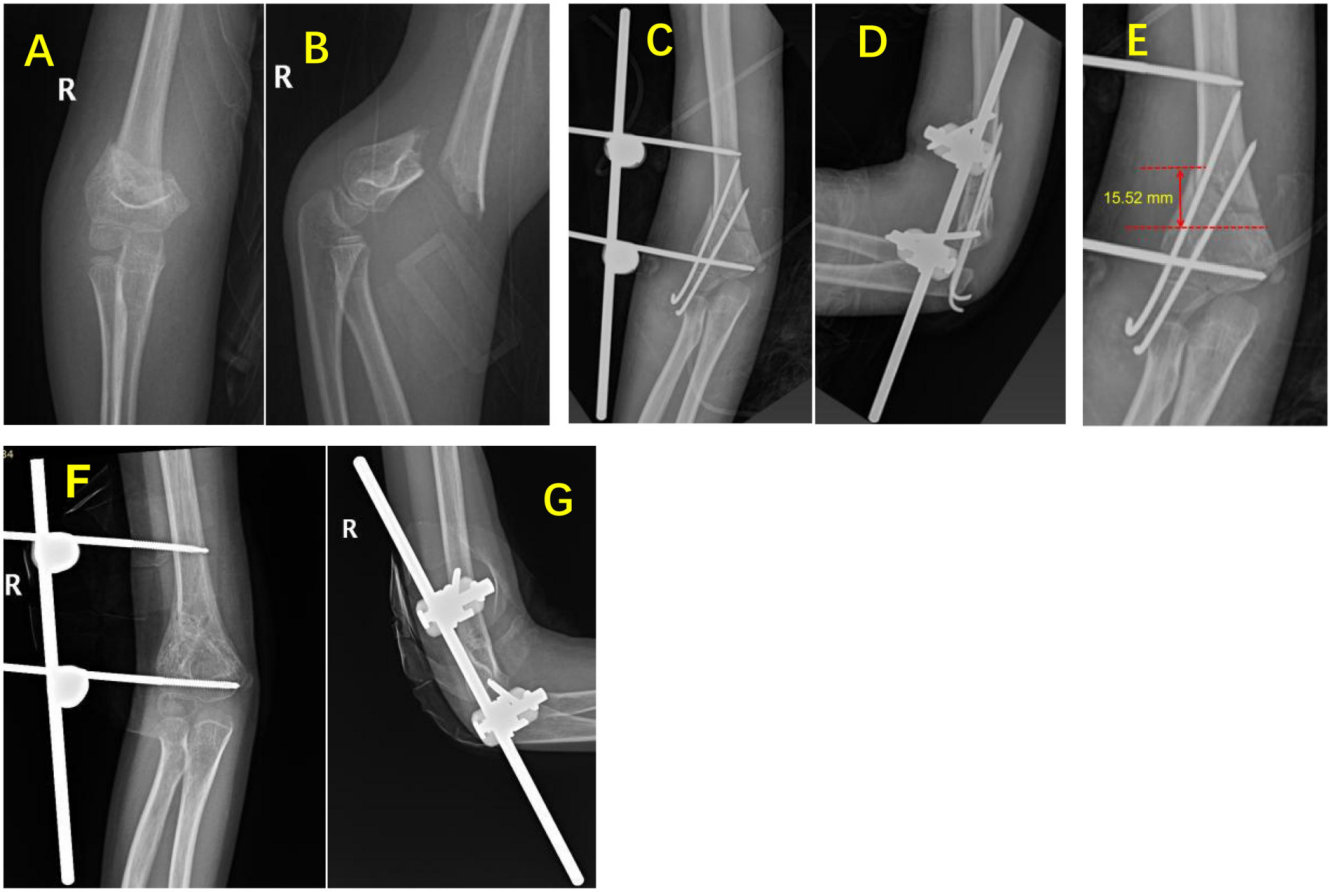
Representative case of linear configuration external fixation. A 6-year-11-month-old girl with right distal humeral MDJ fracture (oblique type). **(A,B)** Preoperative anteroposterior and lateral radiographs showing displaced fracture. **(C,D)** Intraoperative radiographs after closed reduction with linear external fixation plus K-wire augmentation. **(E)** Fracture line height measurement: 15.52 mm. **(F,G)** 12-week follow-up radiographs demonstrating satisfactory union; external fixator removed.

**Figure 3:**
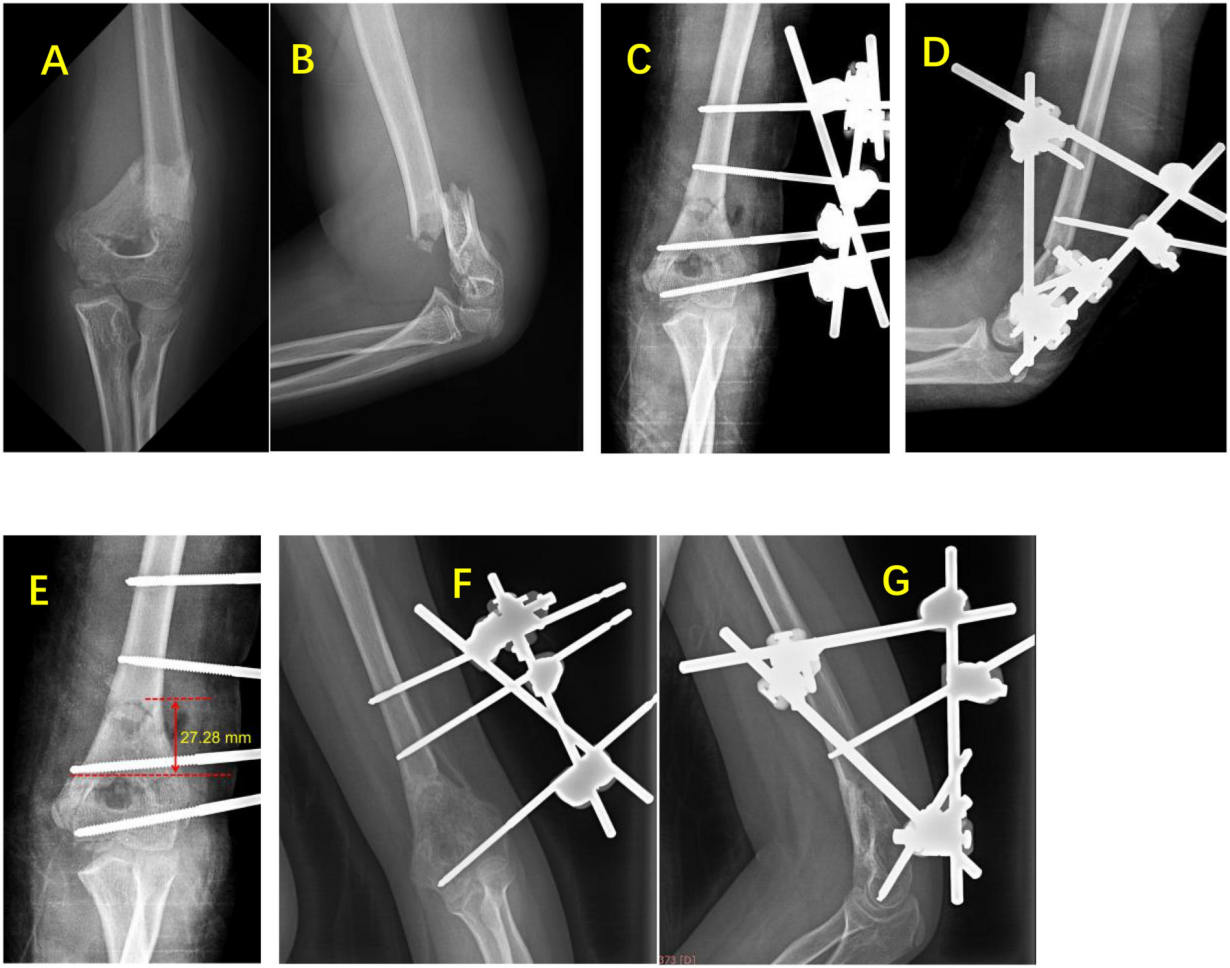
Representative case of triangular configuration external fixation. A 9-year-4-month-old girl with left distal humeral MDJ fracture (transverse type). **(A,B)** Preoperative radiographs. **(C,D)** Intraoperative radiographs after triangular external fixation (no K-wires). **(E)** Fracture line height: 27.28 mm. **(F,G)** 5-month follow-up showing complete union; external fixator removed (one distal half-pin removed at 3 months).

All fractures achieved successful union (Figures 2, 3). The mean fracture healing time was (11.0 ± 2.6) weeks (range: 8.3–14.9 weeks) in the “linear” configuration group and (10.2 ± 3.2) weeks (range: 7.4–21.4 weeks) in the “triangular” configuration group. At the final follow-up, the mean Baumann angle was (72.80 ± 3.71 °) in the “linear” configuration group (range: 65.94–88.01 °) and (73.81 ± 7.40 °) in the “triangular” configuration group (range: 58.62–88.95 °). The mean shaft-condylar angle was (39.38 ± 8.32 °) in the “linear” configuration group (range: 29.05–52.86 °) and (35.51 ± 7.67 °) in the “triangular” configuration group (range: 17.69–46.17 °). There were no statistically significant differences between the two groups for any of these parameters (*P* > 0.05, Table 2). The ICC values demonstrated excellent agreement between the two independent radiologists: Baumann angle ICC = 0.91 (95% CI: 0.84–0.95), Shaft-Condylar Angle ICC = 0.89 (95% CI: 0.81–0.94), and fracture line height ICC = 0.94 (95% CI: 0.89–0.97). All ICC values exceeded the pre-specified threshold of 0.85, confirming excellent measurement reliability. For the primary analysis, the mean values of the two observers' measurements were used.

**Table 2:** Comparison of radiographic outcomes between groups.

Group	Healing time (weeks)	Baumann angle (°)	Shaft–condylar angle (°)	Flynn scores	
				Excellent/good	Fair/poor
Linear	11.0 ± 2.6	72.80 ± 3.71	39.38 ± 8.32	17	1
Triangular	10.2 ± 3.2	73.81 ± 7.40	35.51 ± 7.67	25	2
Statistic	*t* = 0.725	*t* = −0.382	*t* = 1.201		
*P* value	0.475	0.706	0.241	1.000^a^	

Data are presented as mean ± standard deviation. mm, millimeters; °, degrees.

a Fisher's exact test for categorical variables with expected cell counts <5.

#### 3.2.2 Functional outcomes

According to the Flynn criteria for elbow function assessment, at the final follow-up, the “linear” configuration group had 15 excellent, 2 good, and 1 fair results, with an excellent-to-good rate of 94.44%. The “triangular” configuration group had 21 excellent, 5 good, and 2 fair results, with an excellent-to-good rate of 92.31%. There was no difference in results between the two groups (*P* > 0.05, Table 2).

#### 3.2.3 Complications

At the final follow-up, complications were recorded. One case in each group showed a decrease in the carrying angle, but without developing cubitus varus deformity. Two cases in each group experienced internal fixation loosening, but this did not affect fracture healing. Seven cases in the “linear” configuration group and eleven cases in the “triangular” configuration group developed mild pin tract infections, all of which improved after wound dressing changes or oral antibiotics. we observed numerically more infections in the triangular configuration group compared to the linear group (11 vs. 7), but the infection rates were similar between the two groups (40.7% vs. 38.9%), suggesting that the structural complexity of the triangular configuration was not a major determinant of infection risk in our study. Neither group experienced nerve injury, fracture redisplacement, or refracture.

#### 3.2.4 Measurement results of fracture line height

Measurements were taken on the anteroposterior view of the elbow joint after fracture reduction (Figures 2E, 3E). The results showed that the average fracture line height was (17.51 ± 3.35) mm (range: 11.82–22.20 mm) in the “linear” configuration group and (28.02 ± 7.93) mm (range: 15.50–40.53 mm) in the “triangular” configuration group.

## 4 Discussion

### 4.1 Efficacy of modular external fixation in treating pediatric distal humerus MDJ fractures

Traditional closed reduction and percutaneous pinning (CRPP) is the gold standard for treating pediatric supracondylar humeral fractures. Both lateral three-pin divergent configuration and crossed medial-lateral pinning can achieve stable fixation (21, 22). However, there is currently no standardized treatment protocol for pediatric distal humerus MDJ fractures. According to previous literature, some scholars tend to follow conventional supracondylar fracture treatment approaches (7–9, 23). A meta-analysis by Aly et al. (10) revealed that closed pinning is associated with prolonged operative time and higher risks of cubitus varus deformity, nerve injury, and loss of elbow function. In our study of 45 patients treated with modular external fixation with or without supplementary K-wires, only 2 cases developed decreased carrying angle, achieving an overall excellent-to-good elbow function rate of 93.33%, with no complications such as nerve injury or fracture redisplacement, demonstrating satisfactory outcomes. As demonstrated by Kamara et al. (5), the cross-sectional area at the MDJ is significantly smaller than that of the supracondylar region, while the longer distal lever arm amplifies rotational and bending moments at the fracture site. Specifically, K-wires inserted from the distal fragment have a shorter bone purchase length and reduced angular divergence. In contrast, modular external fixation addresses these biomechanical constraints by: (1) allowing pin placement at optimal locations both proximal and distal to the fracture, thereby maximizing lever arm advantage; (2) providing rigid frame constructs that resist multiplanar deforming forces; and (3) enabling real-time adjustment of fracture alignment during surgery. These biomechanical advantages provide the clinical justification for why external fixation may achieve superior stability compared to CRPP in MDJ fractures.

Elastic stable intramedullary nail (ESIN) is another frequently reported method for treating pediatric distal humerus MDJ fractures besides K-wire fixation, but there are currently few reports on it. A comparative study by Ge et al. (11) showed that among 19 patients treated with K-wires, 6 developed cubitus varus deformity, whereas only 1 of 20 patients treated with ESIN developed this complication. They identified the fixation method as an independent risk factor for postoperative cubitus varus. Li et al. (24) reported on non-comminuted pediatric distal humerus MDJ fractures treated with either ESIN or K-wires, suggesting that higher fracture lines favor ESIN stability, while lower fracture lines favor K-wire stability. To date, no literature has compared ESIN with modular external fixation. Reviewing our data, there were very few cases treated with ESIN (two cases), therefore we are unable to compare the two methods. However, modular external fixation offers numerous advantages, including versatile spatial configuration, flexible half-pin placement as needed, and the ability to use half-pins to assist fracture reduction. Compared to ESIN, it requires no open approach and eliminates the need for secondary hardware removal surgery. In our study, all patients tolerated clinic removal with topical anesthesia alone, which compares favorably to ESIN removal that universally requires operating room scheduling and general anesthesia.

### 4.2 Technical considerations for applying modular external fixation in pediatric distal humerus MDJ fractures

When applying modular external fixation for pediatric distal humerus fractures, avoiding radial nerve injury is critically important. Based on the fracture line location and radial nerve course, the nerve typically courses anterolaterally around the elbow joint at the distal fracture fragment. Therefore, two half-pins inserted laterally at the elbow joint are positioned within the safe zone and are unlikely to injure the radial nerve. However, the proximal half-pin insertion site is precisely where the radial nerve transitions from posterior to anterior, representing the highest injury risk. Consequently, after making a percutaneous incision, we must perform blunt dissection to the periosteum, position a protective sleeve against the periosteum within the incision tract, and then insert the half-pin. This ensures that the entire pinning process occurs within the sleeve protection, without contact with surrounding soft tissues, effectively preventing iatrogenic radial nerve injury. Meanwhile, when inserting the lateral half-pin into the proximal end of the fracture, we maintained the elbow joint in a semi-flexed position, at which point the radial nerve is relatively relaxed. None of our cases experienced intraoperative radial nerve injury, confirming this method as safe and reliable.

Fracture reduction and fixation constitute the critical surgical components. After satisfactory half-pin placement, the surgeon can manipulate the proximal and distal half-pins while the assistant maintains traction, correcting coronal plane displacement by pushing or pulling the distal fragment medially or laterally. Simultaneously, the distal half-pins can be used to adjust the valgus angle of the fracture fragments to prevent postoperative cubitus varus deformity. After achieving satisfactory coronal plane correction, temporarily lock the external frame and obtain fluoroscopic views of the sagittal plane alignment. Then partially loosen the frame and correct sagittal plane alignment by adjusting the elbow flexion angle combined with manual manipulation. After confirming satisfactory alignment, re-tighten the frame and reconfirm coronal plane alignment. If supplementary K-wires are needed for enhanced stability, continuous traction and reduction of the fracture fragments must be maintained before percutaneous K-wire insertion to prevent loss of alignment.

### 4.3 Configuration selection of modular external fixation based on fracture line height

In the “linear” configuration, both half-pins are inserted on the radial side of the elbow joint, providing excellent control of coronal plane stability. Lateral compression can also be applied as needed to prevent cubitus varus deformity. In terms of biomechanical characteristics, this method is significantly different from traditional simple K-wire fixation. However, this linear structure is fixed to a single plane and cannot achieve three-dimensional stability, especially in terms of rotational stability (5). Adding K-wire fixation can compensate for this drawback and is simple and practical. Reviewing the literature on external fixation for supracondylar humeral fractures in children (16–19), researchers have mostly used radial external fixators, often combined with K-wires to increase stability, similar to the “linear” configuration used in this study. The clinical follow-up results were good in the 18 children treated with this configuration. However, in distal humeral MDJ fractures, the higher the fracture line, the more difficult it is to insert K-wires for combinede fixation, and the poorer the fixation stability (5). But this creates space at the distal end of the fracture for the insertion of a second half-pin to achieve stability. At this point, we insert the second half-pin at the proximal end of the fracture at a 45° angle anterolaterally to the elbow joint. This allows the four half-pins to form a triangular configuration rather than a “linear” configuration, which is a geometrically stable configuration in space, In this study, the remaining 27 cases were fixed using a “triangular” configuration. If the fracture ends were judged to be stable during surgery, there was no need to use K-wires in combination, and the clinical follow-up results were equally good. Currently, there are no similar reports in the literature.

To further facilitate clinical application, we retrospectively measured the fracture line height. The results showed that the average height in the “linear” configuration group was (17.51 ± 3.35) mm, ranging from 11.82 to 22.20 mm, while the average height in the “triangular” configuration group was (28.02 ± 7.93) mm, ranging from 15.50 to 40.53 mm. Therefore, this study proposes a graded treatment strategy based on the fracture line height: low-position MDJ fractures should be treated with a “linear” configuration combined with Kirschner wires, and high-position MDJ fractures should be treated with a “triangular” configuration. Based on our findings and clinical experience, we propose a height-based treatment strategy: low-position MDJ fractures (<20 mm) may be effectively managed with a linear configuration supplemented by K-wires, whereas high-position fractures (≥20 mm) appear well-suited for a triangular configuration, which provides inherent spatial stability without requiring additional K-wire fixation, and there is enough space to insert a second half-pin 1 cm above the olecranon fossa and away from the fracture end. However, this recommendation is derived from retrospective observations and requires validation through prospective biomechanical and clinical studies, future RCTs are essential to validate thisstrategy.

### 4.4 Study limitations

This retrospective clinical study cannot perform randomized grouping before treatment like prospective studies. Additional limitations include: (1) The relatively small overall sample size may introduce bias to the results, and may lead to a Type II error, potentially masking subtle intergroup differences. (2) The follow-up duration was relatively short, with some patients not yet followed until skeletal maturity, precluding assessment of long-term remodeling and growth disturbances. (3) The retrospective design and non-randomized grouping introduce selection bias, as configuration choice was based on surgeon preference and fracture characteristics. (4) Lack of blinding in outcome assessment may introduce detection bias, particularly for subjective measures like Flynn scores. (5) The proposed 20 mm threshold for configuration selection was empirically derived and lacks formal statistical validation through ROC analysis, this cutoff should be considered a preliminary clinical observation requiring prospective and multicenter confirmation.

## 5 Conclusion

Our results suggest that closed reduction with modular external fixator is a viable alternative for treating pediatric distal humeral MDJ fractures that enables early functional exercise, with minimally invasive and achieves favorable elbow function recovery, has minimal complications, and eliminates the need for secondary hardware removal surgery, demonstrating satisfactory clinical outcomes. Low-position MDJ fractures (<20 mm) with a linear configuration can be sufficiently stabilized with K-wires, and the “triangular” configuration is more suitable for high-level fractures (≥20 mm).

## Data Availability

The original contributions presented in the study are included in the article/Supplementary Material, further inquiries can be directed to the corresponding author.

## References

[1] Fayssoux RS Stankovits L Domzalski ME Guille JT. Fractures of the distal humeral metaphyseal-diaphyseal junction in children. J Pediatr Orthop. (2008) 28(2):142–6. 10.1097/BPO.0b013e3181653af3 18388705

[2] Tokyay A Okay E Cansü E Aydemir AN Erol B. Effect of fracture location on rate of conversion to open reduction and clinical outcomes in pediatric gartland type III supracondylar humerus fractures. Ulus Travma Acil Cerrahi Derg. (2022) 28(2):202–8. 10.14744/tjtes.2020.23358 35099030 PMC10443146

[3] Bahk MS Srikumaran U Ain MC Erkula G Leet AI Sargent MC Patterns of pediatric supracondylar humerus fractures. J Pediatr Orthop. (2008) 28(5):493–9. 10.1097/BPO.0b013e31817bb860 18580360

[4] Anari JB Arkader A Spiegel DA Baldwin KD. Approaching unusual pediatric distal humerus fracture patterns. J Am Acad Orthop Surg. (2019) 27(9):301–1. 10.5435/JAAOS-D-17-00326 30480586

[5] Kamara A Ji X Liu T Zhan Y Li J Wang E. A comparative biomechanical study on different fixation techniques in the management of transverse metaphyseal-diaphyseal junction fractures of the distal humerus in children. Int Orthop. (2019) 43(2):411–6. 10.1007/s00264-018-3968-x 29744649

[6] Liu C Kamara A Liu T Yan Y Wang E. Mechanical stability study of three techniques used in the fixation of transverse and oblique metaphyseal-diaphyseal junction fractures of the distal humerus in children: a finite element analysis. J Orthop Surg Res. (2020) 15(1):34. 10.1186/s13018-020-1564-4 32020882 PMC7001280

[7] Sen RK Tripathy SK Kumar A Agarwal A Aggarwal S Dhatt S. Metaphyseo-diaphyseal junction fracture of distal humerus in children. J Pediatr Orthop B. (2012) 21(2):109–14. 10.1097/BPB.0b013e32834ba9d6 21897299

[8] Qureshi SA Ahmad I Masood F Raza GH Nadeem RD Nadeem A Short term outcome of treatment with percutaneous cross kirschner wires in paediatric distal humeral metaphyseal diaphyseal junction fractures. J Fatima Jinnah Med Univ. (2022) 16:79–83. 10.37018/SAQI4789

[9] Park MS Kim JR Sung KH Moon YJ Lee SC Wang SI. Comparison of functional and cosmetic outcomes according to fracture level in gartland type III pediatric supracondylar humerus fractures. Clin Orthop Surg. (2023) 15(4):668–77. 10.4055/cios22220 37529183 PMC10375807

[10] Aly AS Mohamed AM Kersh A A M. Management of pediatric distal humerus metaphyseal-diaphyseal junction fracture: a systematic review and meta-analysis. J Child Orthop. (2024) 18(4):421–31. 10.1177/18632521241262169 39100985 PMC11295369

[11] Ge YH Wang ZG Cai HQ Yang J Xu YL Li YC. Flexible intramedullary nailing had better outcomes than kirschner wire fixation in children with distal humeral metaphyseal-diaphyseal junction fracture: a retrospective observational analysis. Int J Clin Exp Med. (2014) 7(10):3568–72. 25419399 PMC4238475

[12] Marengo L Canavese F Cravino M De Rosa V Rousset M Samba A Outcome of displaced fractures of the distal metaphyseal-diaphyseal junction of the humerus in children treated with elastic stable intramedullary nails. J Pediatr Orthop. (2015) 35(6):611–6. 10.1097/BPO.0000000000000340 25379828

[13] Dong Z Zhang ZQ Tang K Lou Y Lin G Sun XS Clinical effect analysis of retrograde elastic stable intramedullary nailing in distal humerus metaphysis-diaphyseal junction fractures in children. Chin J Appl Clin Pediatr. (2020) 35(14):1089–92. 10.3760/cma.j.cn101070-20191210-01228

[14] Sénès FM Catena N. Intramedullary osteosynthesis for metaphyseal and diaphyseal humeral fractures in developmental age. J Pediatr Orthop B. (2012) 21(4):300–4. 10.1097/BPB.0b013e328353d96d 22555378

[15] Greve F Biberthaler P Castellani C Singer G Till H Wegmann H. Beneficial perioperative aspects favor the use of percutaneous crossed pinning over antegrade nailing in pediatric supracondylar fractures-A retrospective comparative study. Children (Basel). (2023) 10(5):830. 10.3390/children10050830 37238378 PMC10216984

[16] Li J Rai S Tang X Ze R Liu R Hong P. Surgical management of delayed gartland type III supracondylar humeral fractures in children: a retrospective comparison of radial external fixator and crossed pinning. Medicine (Baltimore). (2020) 99(10):e19449. 10.1097/MD.0000000000019449 32150100 PMC7478454

[17] Horst M Altermatt S Weber DM Weil R Ramseier LE. Pitfalls of lateral external fixation for supracondylar humeral fractures in children. Eur J Trauma Emerg Surg. (2011) 37(4):405–10. 10.1007/s00068-010-0062-5 26815277

[18] Gris M Van Nieuwenhove O Gehanne C Quintin J Burny F. Treatment of supracondylar humeral fractures in children using external fixation. Orthopedics. (2004) 27(11):1146–50. 10.3928/0147-7447-20041101-08 15566124

[19] Slongo T Schmid T Wilkins K Joeris A. Lateral external fixation–a new surgical technique for displaced unreducible supracondylar humeral fractures in children. J Bone Joint Surg Am. (2008) 90(8):1690–7. 10.2106/JBJS.G.00528 18676899

[20] Flynn JC Matthews JG Benoit RL. Blind pinning of displaced supracondylar fractures of the humerus in children. Sixteen years’ experience with long-term follow-up. J Bone Joint Surg Am. (1974) 56(2):263–72. 4375679

[21] Afaque SF Singh A Maharjan R Ranjan R Panda AK Mishra A. Comparison of clinic-radiological outcome of cross pinning versus lateral pinning for displaced supracondylar fracture of humerus in children: a randomized controlled trial. J Clin Orthop Trauma. (2020) 11(2):259–63. 10.1016/j.jcot.2019.01.013 32099290 PMC7026539

[22] Eguia F Gottlich C Lobaton G Vora M Sponseller PD Lee RJ. Mid-term patient-reported outcomes after lateral versus crossed pinning of pediatric supracondylar humerus fractures. J Pediatr Orthop. (2020) 40(7):323–8. 10.1097/BPO.0000000000001558 32271317

[23] Zhou H Zhang G Li M Liu X Qu X Cao Y Closed reduction and percutaneous pinning in the treatment of humeral distal metaphyseal-diaphyseal junction fractures in children: a technique note and preliminary results. Front Pediatr. (2021) 9:670164. 10.3389/fped.2021 34222144 PMC8247651

[24] Li M Liu T Li Q Li L Zhang L Shi L Wang E. Fixation for metaphyseal-diaphyseal junction noncomminuted fracture of the distal humerus in children: k-wire or ESIN, how to decide? Front Pediatr. (2025) 13:1640764. 10.3389/fped.2025.1640764 40843076 PMC12364858

